# Discovery of Diverse Rodent and Bat Pestiviruses With Distinct Genomic and Phylogenetic Characteristics in Several Chinese Provinces

**DOI:** 10.3389/fmicb.2018.02562

**Published:** 2018-10-24

**Authors:** Zhiqiang Wu, Bo Liu, Jiang Du, Junpeng Zhang, Liang Lu, Guangjian Zhu, Yelin Han, Haoxiang Su, Li Yang, Shuyi Zhang, Qiyong Liu, Qi Jin

**Affiliations:** ^1^MOH Key Laboratory of Systems Biology of Pathogens, Institute of Pathogen Biology, Chinese Academy of Medical Sciences & Peking Union Medical College, Beijing, China; ^2^Key Laboratory of Zoonosis of Liaoning Province, College of Animal Science and Veterinary Medicine, Shenyang Agricultural University, Shenyang, China; ^3^State Key Laboratory for Infectious Diseases Prevention and Control, National Institute for Communicable Disease Control and Prevention, Chinese Center for Disease Control and Prevention, Beijing, China; ^4^EcoHealth Alliance, New York, NY, United States

**Keywords:** pestivirus, bats, rodents, viral evolution, viral disease emergence

## Abstract

Bats and rodents are widely distributed worldwide and can be native or intermediate reservoirs of many important zoonotic viruses. Pestiviruses are a group of virus species of the genus *Pestivirus* under the family *Flaviviridae* that can infect a wide variety of artiodactylous hosts, including swine and ruminants. Two classic types of pestiviruses, bovine viral diarrhea virus and classical swine fever virus, are important causative agents of mild-to-severe disease in bovine and swine hosts, respectively, and cause tremendous economic losses in these industries. Recent reports revealed that bats and rodents could also act as natural hosts of pestiviruses and an atypical porcine pestivirus, which cause disease in piglets, showed a close genetic relationship with a specific bat pestivirus, RaPestV-1. This study aimed to describe the detection and characterization of novel pestiviruses from bats and rodents in different locations by analyzing the available bat and rodent virome data from throughout China. Two bat pestivirus species and four rodent pestivirus species that are distinct from other known viruses were identified and sequenced. These viruses were identified from two bat species and four rodent species in different Chinese provinces. There were two distinct lineages present in these viruses, that differ from artiodactylous pestivirus. These findings expand our understanding of the genetic diversity of pestiviruses in bats and rodents and suggest the presence of a diverse set of pestiviruses in non-artiodactylous hosts. This study may provide new insight for the prevention of future viral disease outbreaks originating from bats and rodents.

## Introduction

The genus *Pestivirus* is contained within the family *Flaviviridae*, which includes positive, single-stranded RNA viruses with ∼12 kb genomes and a single open reading frame that encodes a polyprotein flanked by 5^′^- and 3^′^- untranslated regions (UTRs). The polyprotein is translated from genomic RNA and translation is initiated by a cap-independent mechanism through a type IV internal ribosomal entry site within the 5^′^-UTR. The single polyprotein is then cleaved into four structural and eight non-structural proteins by cellular and viral proteases. Pestiviruses were previously divided into four species, including bovine viral diarrhea viruses 1 and 2 (BVDV-1 and BVDV-2), classical swine fever virus (CSFV), and border disease virus (BDV) of sheep or goats (Becher et al., 2003; Simmonds et al., 2017). More recently, the original four species were re-classified as *Pestivirus A, Pestivirus B, Pestivirus C*, and *Pestivirus D*. Since several new pestiviruses have been characterized in other hosts, seven new species of *Pestivirus* were identified: *Pestivirus E* (pronghorn antelope pestivirus), *Pestivirus F* (porcine Bungowannah virus), *Pestivirus G* (giraffe pestivirus), *Pestivirus H* (Hobi-like bovine pestivirus), *Pestivirus I* (sheep Aydin-like pestivirus), *Pestivirus J* (rat pestivirus), and *Pestivirus K* (atypical porcine pestivirus, APPV) (Avalos-Ramirez et al., 2001; Kirkland et al., 2007; Becher et al., 2012; Liu et al., 2012; Firth et al., 2014; Hause et al., 2015; de Groof et al., 2016; Smith et al., 2017).

Pestiviruses can infect a variety of artiodactylous hosts, including swine and ruminants (Tautz et al., 2015). Diseases associated with pestiviruses include hemorrhagic symptoms, abortion, respiratory disease, and fatal mucosal disease. BVDV and CSFV are severely pathogenic in cattle and swine, respectively, and are endemic in many countries (Liu et al., 2012; Chander et al., 2014). Recently, the divergent pestiviruses, Bungowannah virus and APPV, began to be considered a threat to pig health (Kirkland et al., 2007; Hause et al., 2015). In previous work from our lab, we found partial RaPestV-1 genome sequence in *Rhinolophus* bats from Hainan province in China. As such, we were the first to describe the identification of pestivirus in species outside the order Artiodactyla (Wu et al., 2012). An RaPestV-1 related pestivirus, APPV, was recently discovered in North America and Europe, and was highly suspected to be the causative agent of a type A-II congenital tremor (CT A-II) in piglets (Hause et al., 2015; de Groof et al., 2016; Postel et al., 2016; Beer et al., 2017; Lamp et al., 2017). Recently, the identification of a divergent pestivirus (Norway rat pestivirus, NRPV) in New York City suggested that Norwegian rats (*Rattus norvegicus*) can also serve as natural hosts for pestivirus (Firth et al., 2014). These findings suggested a wider pestivirus host range than previously known and highlighted the need for an improved understanding of the biodiversity and evolution of pestivirus in bats and rodents.

Bats (Order *Chiroptera*) and rodents (Order *Rodentina*) are two of the most widely geographically distributed mammals and display the most extensive species diversity (Wilson and Reeder, 2005). They are also considered to be major natural hosts of a large variety of viruses, including many important zoonotic viruses that can cause severe diseases in humans and domestic animals, including henipaviruses, severe acute respiratory syndrome coronavirus, hantaviruses, and arenaviruses (Wang et al., 2006; Meerburg et al., 2009; Smith and Wang, 2013; Yang et al., 2013; Han et al., 2015; Gao et al., 2016; Wu et al., 2018a). In the present study, we use virome analysis by using sequence-independent PCR amplification, next-generation sequencing, and sequence similarity comparisons to classify identified pestiviruses. Novel sequencing reads related to pestivirus were found in samples collected from different bat and rodent species. Genomic and phylogenetic analyses of these viruses revealed the presences of six novel pestivirus species in bat and rodent hosts.

## Materials and Methods

### Ethics Statement

Bats and rodents were treated in accordance with the guidelines outlined in the Regulations for the Administration of Laboratory Animals (Decree No. 2 of the State Science and Technology Commission of the People’s Republic of China, 1988). The sampling was approved by the Ethics Committee of the Institute of Pathogen Biology, Chinese Academy of Medical Sciences & Peking Union Medical College (Approval number: IPB EC20100415).

### Animal Samples

A total of 4,511 individuals bats across 40 species and 2,752 rodents covering 50 species were sampled by obtaining both pharyngeal and anal swabs between October 2010 and July 2017 throughout China (Wu et al., 2016, 2018b). All bats and rodents collected in this study were considered to be apparently healthy and showed no overt signs of disease. Pharyngeal and anal swab samples from captured bats and rodents were immersed in virus sampling tubes (Yocon, China) containing maintenance medium and temporarily stored at -20°C. The samples were then transported to the laboratory and stored at -80°C.

### Viral Nucleic Acid Library Construction and Next-Generation Sequencing

The samples from each species were pooled by adding 1 ml from each maintenance media sample into a fresh sample tube. The pooled samples were classified by species, and then processed using a viral particle-protected, nucleic acid purification method as described in our previous studies (Wu et al., 2012, 2016). The extracted RNA and DNA were amplified using sequence-independent PCR. Amplified viral nucleic acid libraries were then sequenced using an Illumina HiSeq2500 sequencer with single, 81–100 bp reads. The raw sequence reads were then filtered using previously described criteria to obtain valid sequences (Wu et al., 2012, 2016; Li et al., 2015a).

### Taxonomic Assignment

Sequence similarity-based taxonomic assignments were conducted as described in our previous study (Wu et al., 2016). Valid sequence reads were aligned to sequences obtained from the NCBI non-redundant nucleotide database (NT) and non-redundant protein database (NR) using BLASTn and BLASTx, respectively. The taxonomies of the aligned reads with the best BLAST scores (E score < 10^-5^) were parsed using the MEGAN 5–MetaGenome Analyzer (Huson et al., 2016).

### Viral Prevalence and Genome Sequencing

Sequence reads classified into the same virus family or genus with MEGAN 5 were extracted. The accurate loci of each reads and the relative distances between reads of the same virus were determined based on the alignment results obtained with MEGAN 5. We designed specific nested primers based on alignment results for RT-PCR to screen individual samples for the presence and prevalence of pestivirus.

The located reads and contigs were used for reads-based PCR to identify partial genomes in positive individual samples (The primer sequences are available in Supplementary Table 1). Based on partial genomic sequences of each virus, the remaining genomic sequences were determined with genome walking, and 5^′^ and 3^′^ rapid amplification of cDNA ends (RACE) by using genome walking kit (TaKaRa), 5^′^ RACE kit (Invitrogen), and 3^′^ full RACE core set, version 2.0 (TaKaRa).

### Genomic and Phylogenetic Analysis

The polyproteins were deduced by comparing them with the sequences in other pestiviruses. The conserved protein families and domains were predicted using either Pfam and InterProScan 5^1^ or the Conserved Domain Database of NCBI. Routine sequence alignments were performed using Clustal Omega, Needle^2^, MegAlign (Lasergene), and T-coffee with manual curation. MEGA6.0 was used to align nucleotide sequences and deduced amino acid sequences using the MUSCLE package with the default parameters (Tamura et al., 2013). The best substitution model (LG + G for polyprotein, LG + G for Npro, JTT + G for Erns, and LG + G + I for NS3) was then evaluated using the Model Selection package. Finally, a maximum-likelihood method with an appropriate model was used to conduct phylogenetic analyses with 1,000 bootstrap replicates (Tamura et al., 2013).

### Nucleotide Sequence Accession Numbers

All genome sequences have been submitted to GenBank. The accession numbers for the four bat pestiviruses are MH282908–MH282911. The accession numbers for the five rodent pestiviruses are KY370099–KY370103. The GA II sequence data have been deposited into the NCBI sequence reads archive (SRA) under accession numbers SRA051252 and PRJNA375958.

## Results

### Virome and Prevalence Analysis for Pestiviruses

**FIGURE 1:**
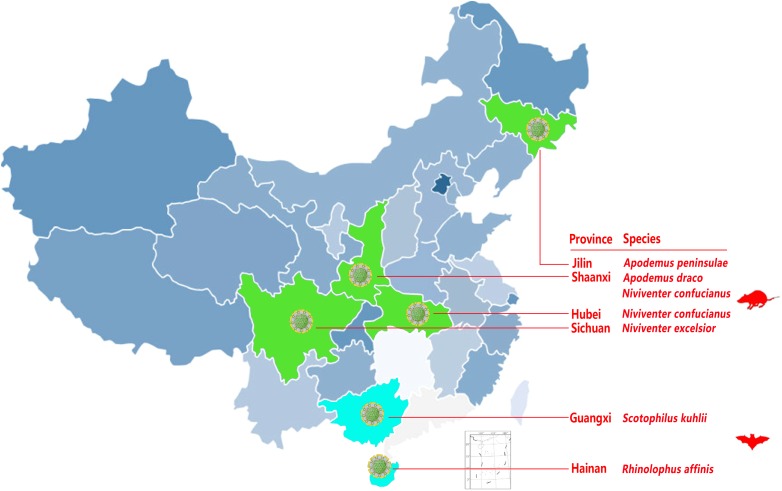
Occurrence of pestivirus-related reads in bats and rodents from different locations. Provinces with pestivirus-related reads are labeled in green and aqua and the animal species from each province is noted in red to the right of the map.

A systematic survey of pestiviruses was performed using small mammal virome data obtained throughout China from October 2010 to May 2017 and indicated the presence of diverse pestiviruses in the mammals tested (Wu et al., 2016, 2018b). A total of 15,164 sequence reads of 81–100 bp were classified into the genus *Pestivirus* based on the NR alignment results generated with MEGAN5 (Wu et al., 2016). These reads showed 31–68% amino acid (aa) identity with known pestiviruses in the GenBank database. Two bat species identified from two provinces and four rodent species from four provinces were found to be pestivirus-positive (Figure 1 and Table 1). Besides the previously reported RaPestV-1 identified from samples of *Rhinolophus affinis* bats in Haikou city of Hainan province, all other bat-derived reads related to pestivirus were only found in samples from *Scotophilus kuhlii* bats in the cities of Nanning and Beihai in Guangxi province. However, reads related to pestivirus were found in samples collected from multiple rodent species, including those in the subfamily *Murinae* taken from four different locations, including *Niviventer excelsior* in Ganzi Tibetan Autonomous Region of Sichuan, *Apodemus peninsulae* in the Korean Autonomous Prefecture of Yanbian of Jilin, *Apodemus draco* in Ankang, Shaanxi, and *Niviventer confucianus* in Wuhan, Hubei as well as in Hanzhong, Shaanxi. The prevalence of specific pestiviruses were then re-evaluated by single-strain screening with specific reads-based nested-PCR, as shown in Table 1. By this method, five bat anal swab samples, twelve rodent anal swab samples, and two rodent pharyngeal swab samples were confirmed to be pestivirus-positive. Two rodent individuals were pestivirus-positive in both pharyngeal and anal swab samples.

**Table 1:** Presence and abundance of pestiviruses from different animal species and locations.

	Species	Province	City	Collection date [yr.mo]	Virus detection rate (pos./indiv. [%])	Positive sample type (pharyngeal/anal)	Virus
Bat	*Scotophilus kuhlii*	Guangxi	Nanning, Beihai	2017.05	4/137 [2.92%]	0/4	BtSk-PV-(1–4)/GX2017
	*Rhinolophus affinis*	Hainan	Haikou	2010.12	1/19 [5.26%]	0/1	RaPestV-1
Rodent	*Niviventer excelsior*	Sichuan	Ganzi	2014.07	1/42 [2.38%]	1/1	RtNe-PV/SC2014
	*Niviventer confucianus*	Hubei	Wuhan	2014.12	3/51 [5.88%]	0/3	RtNc-PV/HuB2014
	*Niviventer confucianus*	Shaanxi	Hanzhong	2015.6	2/50 [4.00%]	0/2	RtNc-PV/SAX2015
	*Apodemus peninsulae*	Jilin	Yanbian	2014.8	2/22 [9.09%]	1/2	RtAp-PV/JL2014
	*Apodemus draco*	Shaanxi	Ankang	2015.6	4/47 [8.51%]	0/4	RtAd-PV/SAX2015

### Genome Organization of Bat Pestiviruses (BPVs)

**FIGURE 2:**
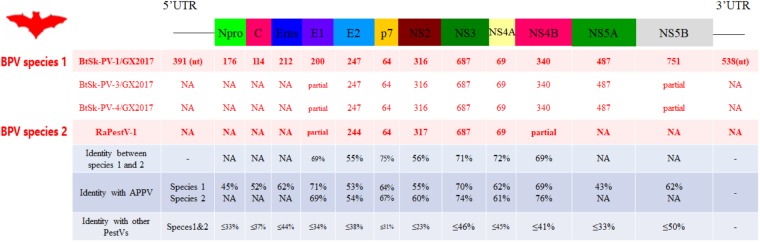
Genomic analysis of BPVs. The genomic organization and 12 mature peptides of BPVs were predicted and shown in the upper diagram. The lengths of the predicted mature peptides and the pairwise amino acid identities (%) of those peptides compared to other *Pestivirus* members are shown in the lower table.

The genome sequences of four viral strains identified from pestivirus-positive *S. kuhlii* bats in Sichuan were characterized (Figure 2). The BPV strains were described as follows: the full-length genome of strain BtSk-PV-1/GX2017 was sequenced and yielded an 11,921 nt genome that encoded a 3,618 aa polyprotein. Strains BtSk-PV-2/GX2017, BtSk-PV-3/GX2017, and BtSk-PV-4/GX2017 were partially sequenced, obtaining partial genome lengths of 680, 7266, and 7132 nt, respectively, that shared 97–98% nt identity between one another and with BtSk-PV-1/GX2017. The polyprotein of these four pestiviruses shared 65% aa identity with RaPestV-1, 59% aa identity with APPV from *Pestivirus K*, and <35% aa identity with pestiviruses of rodents and other hosts. According to the general rules of taxonomy proposal accepted by ICTV (Smith et al., 2017), The four identified BPVs could be defined as BPV species 1, and the previously partially sequenced strain, RaPestV-1, was defined as BPV species 2.

The 5^′^- UTR, 12 mature pestivirus peptides, and 3^′^- UTR of BPVs were predicted by sequence alignment with available pestivirus reference genomes and known cleavage sites, which have been determined previously in APPV and other artiodactylous pestiviruses (Figure 2). For BPV species 1, we used strain BtSk-PV-1/GX2017 as a reference, for which the 5^′^- UTR was 391 nt and the 3^′^- UTR was 538 nt. Each of these are consistent with the lengths of other pestivirus 5^′^- and 3^′^- UTR’s. Each peptide of BPV species 1 and 2 aligned best with those of APPV, even when the overall identities between BPV species 1 and APPV were relatively low when compared with the identities between BPV species 2 and APPV. Each mature peptide had very low aa sequence identity when compared with those of other pestiviruses. The non-structural autoprotease (Npro) protein of BPV species 1 was predicted to be self-cleaved from the polyprotein between Cys176 and Ser177, and the conserved catalytic residues were identified as Glu42, His67, and Cys87. A slightly larger core protein (C) was located between Npro and the three envelope (E) glycoproteins (Erns, E1, and E2). An RNase T2 superfamily domain with uridylate specificity was identified in Erns between aa 320 and 374 of the polyprotein. A peptidase S31 domain between aa 1330 and 1508, and a helicase associated domain between aa 1557 and 1875 were identified in the NS3 protease. Due to the lack of significant similarity to other pestiviruses, some domains that were conserved in previously reported artiodactylous pestiviruses were not found in the polypeptides of BPV species 1 and 2.

### Genome Organization of Rodent Pestiviruses (RPVs)

**FIGURE 3:**
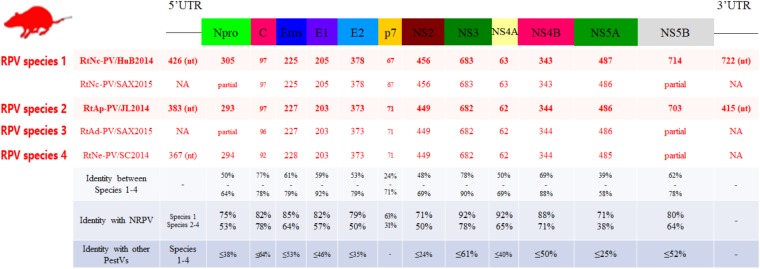
Genomic analysis of RPVs. The genomic organization and 12 mature peptides of RPVs were predicted and are shown in the upper diagram. The lengths of predicted mature peptides, and their pairwise amino acid identities (%) compared to other *Pestivirus* members are shown in the lower table.

The genome sequences of five virus strains from representative positive samples were characterized (Figure 3). The strains were described as follows: strain RtAp-PV/JL2014, identified in *A. peninsulae* from Jilin had a 12,768 nt genome, encoding a 3,989 aa polyprotein. RtNc-PV/HuB2014, which was found in *N. confucianus* from Hubei had a 13,220 nt genome, encoding a 4,023 aa polyprotein. RtAd-PV/SAX2015 in *A. draco* from Shaanxi had a partially sequenced genome, of 11,551 nt, which encoded a 3,850 aa polyprotein, denoted as a partial polyprotein. RtNe-PV/SC2014 in *N. excelsior* from Sichuan had an 11,644 nt partially sequenced genome, encoding a 3,759 aa partial polyprotein, and lastly, we identified RtNc-PV/SAX2015 in *N. confucianus* from Shaanxi with an 11,435 nt partially sequenced genome with a 3,811 aa partial polyprotein. We propose the name Rodent pestivirus (RPV) for these five pestivirus strains.

The five RPVs shared similar genome sizes with one other and were each larger than all known PVs from artiodactylous hosts (Figure 3). The polyproteins encoded by three of the five RPVs (RtAp-PV/JL2014, RtAd-PV/SAX2015, and RtNe-PV/SC2014) had 68, 69, and 77% aa identities to each other, respectively, while they had only 59% aa identity to that of NRPV, and <39% aa identity to those of BPVs and other pestiviruses. The polyproteins of the remaining two RPVs (RtNc-PV/HuB2014 and RtNc-PV/SAX2015) showed 93% aa identity to each other, 80% aa identity to that of NRPV, and <40% aa identity to BPVs and other pestiviruses. According to the ICTV criteria, the five identified RPVs could be further classified into two four species, named as RPV species 1 (RtNc-PV/HuB2014 and RtNc-PV/SAX2015), species 2 (RtAp-PV/JL2014), species 3 (RtAd-PV/SAX2015), and species 4 (RtNe-PV/SC2014), with only 59–77% intergroup aa identities.

The 5^′^- UTR, 12 mature pestivirus peptides, and 3^′^- UTR of RPVs were predicted by aa alignment to pestivirus reference genomes and known cleavage sites, as determined in NRPV and other artiodactylous pestiviruses. The aa identity of mature peptides between RPVs and other pestiviruses were then calculated from pairwise alignments (Figure 3). Despite the extensive sequence diversity, each peptide of RPV species 1 matched best with that of NRPV, the identities between RPV species 1 and NRPV were higher than between RPV species 2–4 and NRPV. The P7 peptides were the most divergent regions of RPVs, as they showed only 31–63% aa identity with P7 of NRPV, respectively, and lacked significant similarity to any other proteins in GenBank. The Npro proteins were 293–304 aa, which is longer than those found in other pestiviruses. The conserved Npro catalytic residues were identified in both RPVs (RtNc-PV/HuB2014: Glu149, His190, and Cys208; RtAp-PV/JL2014: Glu133, His179, and Cys197; RtNe-PV/SC2014: Glu138, His180, and Cys198), but the self-cleavage site for Npro of RPV species 2–4 is between Cys and Asn (RtAp-PV/JL2014 and RtNe-PV/SC2014) or between Cys and Glu (RtAd-PV/SAX2015), which differs from those of RPV species 1 and other pestiviruses. The RNase T2 superfamily domains in Erns and peptidase S31 domains and helicase associated domains in the NS3 protease were also identified in RPVs.

### Phylogenetic Analysis

**FIGURE 4:**
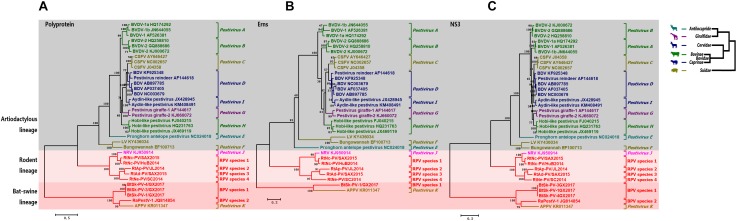
Phylogenetic analysis. Phylogenetic trees based on pestivirus **(A)** partial polyprotein, **(B)** Erns, and **(C)** NS3 were constructed (left). All BPVs and RPVs are labeled in red font. The evolutionary lineages of involved artiodactylous hosts (right) were drawn from genus to family according to previous reports (Jansa and Weksler, 2004; Meredith et al., 2011), viruses of different hosts are labeled in different colors.

Phylogenetic trees based on the aa sequences of each strain’s partial polyprotein, Erns, and NS3 were used to describe the evolutionary relationships between pestiviruses. Consistent with the phylogeny of their hosts in order level, all pestiviruses were divided into three main lineages; the artiodactylous lineage, bat-swine lineage, and rodent lineage (Figure 4). Most pestiviruses that are associated with animals of the families *Suidae, Bovidae, Cervidae, Antilocapridae*, and *Giraffidae* under the order *Artiodactyla* clustered together under the artiodactylous lineage. However, viruses under the artiodactylous lineage tend to be inconsistent with host phylogenies, and multiple incongruous relationships between the phylogenies of hosts and viruses were identified. For example, diverse bovine and swine pestiviruses were scattered in the genus *Pestivirus* with much higher genetic diversity than pestiviruses of other host species.

In accordance with pair-wise similarity analysis, BPVs identified from animals of the families *Vespertilionidae* and *Rhinolophidae* were classified into BPV species 1 and 2 in the bat lineage, respectively, while RPVs identified from animals of the subfamily *Murinae* were classified into RPV species 1–4 in the rodent lineage. The only exception was APPV. This viral species was identified in pigs but formed a distinct lineage located outside the artiodactylous lineage, but instead, was associated within the bat-swine lineage.

## Discussion

Bats and rodents are the most abundant and diverse mammals in the world. They often live in close contact with human populations and their domestic animals, and act as a bridge between humans, domestic animals, and other wildlife, thus exposing humans to some zoonoses in these natural ecosystems (Meerburg et al., 2009; Smith and Wang, 2013; Chen et al., 2014, 2017; Han et al., 2015; Olival et al., 2017). Many viral causative agents of human and domestic animal diseases, including severe respiratory syndrome coronavirus, henipaviruses, hantaviruses, and arenaviruses, are widely recognized as having originated or been transmitted from wild bat or rodent hosts (Wang et al., 2006; Guo et al., 2013; Wu et al., 2014; Li et al., 2015b; Blasdell et al., 2016). Recently, a novel causative agent of fatal disease in pigs known as swine acute diarrhea syndrome coronavirus, was confirmed as being an HKU2-related alphacoronavirus that originated in horseshoe bats (Rhinolophus spp.) (Zhou et al., 2018). Furthermore, an atypical porcine pestivirus, APPV, which caused piglet CT A-II in Europe and North America showed close resemblance to a horseshoe bat pestivirus, RaPestV-1 (Hause et al., 2015; de Groof et al., 2016; Postel et al., 2016; Beer et al., 2017). The identification of diverse rodent arteriviruses in our recent report also revealed a much closer common ancestor of porcine reproductive and respiratory syndrome virus in the rodents pestiviruses (Wu et al., 2018b). These new findings further emphasize the importance of bats and rodents as natural reservoirs of viruses that threaten the health of pigs and by extension, cause tremendous economic losses to the swine industry. Though a large volume of wildlife virome data has been obtained in the past decade, a predictive analysis revealed that the viral richness in wildlife is somewhat limited, and there is still a large number of “missing potentially zoonotic viruses.” This missing data merits further systematic global surveillance, especially in bats, rodents, and primates (Olival et al., 2017).

This study described the identification of novel BPVs and RPVs in different bat and rodent species across several Chinese regions, which revealed that these two mammals served as natural hosts for pestiviruses. Though the virome analyses were conducted on highly diverse samples of bat and rodent species throughout the country, only two bat species in two provinces and four rodent species from four provinces were found to be pestivirus positive. This suggested that RPVs and BPVs are not as broadly distributed across hosts and geographical distance as other bat- or rodent- borne RNA viruses such as coronaviruses, picornaviruses, and hantaviruses (Guo et al., 2013; Du et al., 2016; Wu et al., 2016). The hosts for BPVs can only be found in south China, while RPVs showed much broader host and geographical distributions then RPVs, with rodents in north, central, and west China serving as natural hosts of RPVs.

The newly identified BPVs and RPVs potentially represented six new species under the genus *Pestivirus*. Considering their divergent phylogenetic positions, different genome sizes and structures, and the minimal sequence similarities of BPVs and RPVs when compared to other pestiviruses, we propose classifying members of the genus *Pestivirus* into three main lineages; bat-swine, rodent, and artiodactylous lineages, based on the order level of their hosts. BPV species 1 and 2, and APPV represent the preliminary species under the bat-swine lineage, and RPV species 1–4 represent the preliminary species under the rodent lineage. Furthermore, the close relationship between APPV and BPVs indicates the presence of a much closer common ancestor of APPV in bats than in other swine pestiviruses. These viruses may have evolved independently in pigs and bats, eventually forming at least three sub-lineages under bat-swine lineage. Given that pestiviruses can cross species barriers to infect a wide range of artiodactylous animals (Becher et al., 1997), further work should be conducted to investigate the prevalence of BPVs and RPVs worldwide and to evaluate their virulence in animal experiments.

## Author Contributions

ZW and QJ conceived the experiments, analyzed the results, and wrote the manuscript. BL, JD, YH, HS, LY, SZ, and QL conducted the experiments and analyzed the results. JZ, LL, and GZ collected the specimens. All the authors reviewed the manuscript.

## Conflict of Interest Statement

The authors declare that the research was conducted in the absence of any commercial or financial relationships that could be construed as a potential conflict of interest.

## Footnotes

1 http://www.ebi.ac.uk/services/proteins

2 http://www.ebi.ac.uk/Tools/
