# Supplementary material for: Discovery of Diverse Rodent and Bat Pestiviruses With Distinct Genomic and Phylogenetic Characteristics in Several Chinese Provinces
Source: Front Microbiol. 2018 Oct 24;9:2562. doi: 10.3389/fmicb.2018.02562 (PMC6207626; doi:10.3389/fmicb.2018.02562)
Supplement: Supplementary file 1 [file Table_1.docx]

**Supplementary Table 1. Specific primers used in this study**

| Virus | Primer code | Primer Sequence (5’-3’) |
| --- | --- | --- |
| BtSk-PV-1/SC2017 | PV4-6F1 | ATAACTGTGGCTTTGAAGGCTGA |
|  | PV4-6F2 | GGTTTGGGTTACAGGAGAGTGG |
|  | PV4-1R1 | TCCCTCTTGACATAGCTTGTTTGTA |
|  | PV4-1R2 | CCAATGTGGCTAATAGCACGAT |
|  | PV4-1F1 | ATGACACATATGCCTGGTAAGAAAG |
|  | PV4-1F2 | GTTGCAAGAACAACACCATGAAG |
|  | PV4-7R1 | ACTATGCCAAGAGGTTTCAACAAC |
|  | PV4-7R2 | TCTCTCCAGAATTCTTACTTCGACC |
|  | PV4-7F1 | GGACTATGATAGGCTACACAGGCTA |
|  | PV4-7F2 | CAGAAATCATGTCCCTTGGGTC |
|  | PV4-3R1 | GGAAACTTTCCCCTCTGTAATAGAC |
|  | PV4-3R2 | GAGGATCATTACAGCCCTCACTG |
|  | PV4-3F1 | CTTGTTAGAAGGAATTCAGACATGC |
|  | PV4-3F2 | CAACCCAGTGAGGGCTGTAAT |
|  | PV4-5R1 | TAGCCCTTACCACTGGTAATTTTG |
|  | PV4-5R2 | GTTTGGAATGTTCACTTCTTCCC |
|  | PV4-5F1 | TTTCTCGTCACTGCCAAAGTTG |
|  | PV4-5F2 | GAAGAGGTAGACTACCAAACCTTGG |
|  | PV4-4R1 | TGTTTCTCATATTCTTCACCACTCC |
|  | PV4-4R2 | TATTGGTTGGTGACTACAGAACTCG |
|  | PV4-16F1 | GGATGAATTAGTCGTCTCTGAAACC |
|  | PV4-16F2 | TCTGAAACCAGCACTACCATTACC |
|  | PV4-79F1 | AACCACCAACGGAGTCTTTCTAG |
|  | PV4-79F2 | ATTGGCATTGTAGAAGGTATTCTTG |
|  | PV4-R1 | TGTCGTACATGCCTATGCTTCTG |
|  | PV4-R2 | GCATCCCTTTACAGATATTCGCT |
| BtSk-PV-2/SC2017 | PV3-4F1 | GCTAAGACCTCAAGTGTGGGTAGAT |
|  | PV3-4F2 | GAGATGTAGTATGCAGGAGAAATGG |
|  | PV3-2R1 | ATGAAGACTATGGCTCCAGCTTC |
|  | PV3-2R2 | TTCAGGGTTGTAACAGTAGCAGAGT |
|  | PV3-2F1 | TTCACTCTGCTACTGTTACAACCCT |
|  | PV3-2F2 | CCCTGAAGCAACTAACATAAAAGG |
|  | PV3-1R1 | CTCCTACTTCCTCAATCTCATACCTC |
|  | PV3-1R2 | GGCCGACATTGCTACGAATC |
| BtSk-PV-3/SC2017 | PV12-7F1 | TGGCAGGGACTACACCTAAAGAA |
|  | PV12-7F2 | GAACAAGAAGTAACTGAGCGAAAGA |
|  | PV12-6R1 | GTCGTTAATGCACAGACCTTCG |
|  | PV12-6R2 | ACAACAGGCACCAGAGGACTG |
|  | PV12-6F1 | GTAATAAATGTCGCCCATGCAC |
|  | PV12-6F2 | ATAATAGTTAAGGTGGCGAAAGGG |
|  | PV12-1R1 | CCTCCACTTTAGTCACCTTTGGTA |
|  | PV12-1R2 | TTCCCCAATCATCACCATGTC |
|  | PV12-1F1 | TCAGGAAAGGTATGTGTGGACTTC |
|  | PV12-1F2 | ACCCCTATACAAAATCTGGTTGTCT |
|  | PV12-10R1 | GAGTCTCTCCAGAATTCTTACTTCGA |
|  | PV12-10R2 | TCTTACTTCGACCTCATCCACG |
|  | PV12-10F1 | TGGGGTCATTGACCAGATCAA |
|  | PV12-10F2 | CGAAGCCTTCATCCAGATGAAT |
|  | PV12-13R1 | CCCAACACCTTAGTTGGTACTCAA |
|  | PV12-13R2 | ACTCAAATTCATGTTGGTCTTCACA |
|  | PV12-13F1 | TGGATGTGGAAACCACAGCTC |
|  | PV12-13F2 | ACAGCTCTGCCTATGGGTACATTA |
|  | PV12-5R1 | TAGTTGATGTATCTCTCCCAGGCA |
|  | PV12-5R2 | AGCTCACATTTGGGGTGTTTACT |
| BtSk-PV-4/SC2017 | PV13-1F1 | TCCTTAAGGATGAAAACTCGACAG |
|  | PV13-1F2 | AGCGAATATCTGTAAAGGGATGC |
|  | PV13-2F1 | CTGGTGCAATGTGCCCAATA |
|  | PV13-2F2 | GACAAAATACCAAAAGATGATTCTGG |
|  | PV13-8R1 | ACAACAGGCACCAGAGGACTG |
|  | PV13-8R2 | CAAGGTAGACCAACTCTAAACTCAGG |
|  | PV13-8F1 | TAATAAATGTCGCCCATGCACT |
|  | PV13-8F2 | ATGCACTCCTGAGTTTAGAGTTGGT |
|  | PV13-5R1 | TCTGCTTCATGGTGTTGTTCTTG |
|  | PV13-5R2 | CCTTTCTTACCAGGCATATGTGTC |
|  | PV13-6F1 | ACACCTCCAGGAGTGGTAACAAC |
|  | PV13-6F2 | AGATTGAGGAAGTAGGAGTGGCTAC |
|  | PV13-7R1 | GTTCTGCTGGACCATTTTGTTATAC |
|  | PV13-7R2 | TGGGTGCATCTTTCAAGTATTTG |
| RtAp-PV/JL2014 | 1013L7PVC15-F1 | ATAGGGCCAAAGTTTTACTCCG |
|  | 1013L7PVC15-F2 | GCCAAAGTTTTACTCCGACTATGA |
|  | 1013L7PVC6-R1 | CCCTTGTGTCAAATTGCCTCTT |
|  | 1013L7PVC6-R2 | GTCAAATTGCCTCTTATTGCCTT |
|  | 1013L7PVC6-F1 | GGGTATGTGTTAAACCATCATGGT |
|  | 1013L7PVC6-F2 | GCCATACAGTGCAGAGATAGGGT |
|  | 1013L7PVC11-R1 | GGGCAGACTCCACAGTAGTAATCA |
|  | 1013L7PVC11-R2 | CAGGTAATAAGCAGTCAACAACCAT |
|  | 1013L7PVC11-F1 | TATGACATAAAGGATGAGTTCTGGG |
|  | 1013L7PVC11-F2 | GCCAGTACATGCTCAAGGATAAATA |
|  | 1013L7PVC14-R1 | CATTGGGGTACTCTAGTAGGGTTTC |
|  | 1013L7PVC14-R2 | CACACAGAGTTCTCTGCTTCCATT |
|  | 1013L7PVC14-F1 | AGAGACAATGGAAGCAGAGAACTCT |
|  | 1013L7PVC14-F2 | CAACTCTTTTTTGGGAAAACACC |
|  | 1013L7PVC1-R1 | GTGGCATAGAAGTAGTACGGTGGT |
|  | 1013L7PVC1-R2 | CGGTGGTTGATCCTTTAGCATT |
|  | 1013L7PVC1-F1 | TGGAGGTGTGTAACAACTATTTGCT |
|  | 1013L7PVC1-F2 | AGTGAAGAACTGACACAGCTCACAA |
|  | 1013L7PVC5-R1 | ATACCACTCTAAGTGGGTTCGCA |
|  | 1013L7PVC5-R2 | TCAAAGATGCATGACAGGATCAC |
|  | 1013L7PVC5-F1 | GTTTCACTTGTATTAGGTGTGGGTG |
|  | 1013L7PVC5-F2 | TTTTAGAGCAGAGTGAGGGCAAG |
|  | 1013L7PVC17-R1 | TTGTAGGCATCCAGCCCTGT |
|  | 1013L7PVC17-R2 | CCCTGTGTAACTCAATTTCCTCTTAC |
|  | 1013L7PVC17-F1 | AGAACGTACACTACAAAGAGCCAAA |
|  | 1013L7PVC17-F2 | AAGAGCCAAAAATAGTGGCAGC |
|  | 1013L7PVC2-R1 | TACTGTGTGGCATGTGCAGTCTT |
|  | 1013L7PVC2-R2 | ACCATTTGGTGCATAGACTAATCCT |
|  | 1013L7PV5-5-1 | GGGTGTATAATGTGTCCA |
|  | 1013L7PV5-5-2 | CACTGGCTTGTCACACTTACCTG |
|  | 1013L7PV5-5-3 | AGGGTCGTAGGTCCACTTTTTCT |
|  | 1013L7PV5-3-1 | TATGAAAACCAGGTTGC |
|  | 1013L7PV5-3-2 | TGATGTACCCGTGGAATCCAC |
|  | 1013L7PV5-3-3 | TCCACTGGTTAGAAGAATCTGCTT |
| RtNc-PV/HuB2014 | 150112PeV-C17-F1 | CAACTACCAAAGAGGCTTGTATGTG |
|  | 150112PeV-C17-F2 | CCCAGGTTTTACAGCAGTTACG |
|  | 150112PeV-C12-R1 | AGCAGGATCACACCCTGTAACAC |
|  | 150112PeV-C12-R2 | CAAGAAGCACAGAAAAGCCACA |
|  | 150112PeV-C12-F1 | CCAGACACAGGACAATATCTGAGAA |
|  | 150112PeV-C12-F2 | TTCCTACAAGTGTCTACGTCAATGG |
|  | 150112PeV-C8-R1 | AGCAGTGGTAGGTATTTGAGTAGTCC |
|  | 150112PeV-C8-R2 | TGGTCACTCTGTTCTAGATCAAACC |
|  | 150112PeV-C8-F1 | GGGAGACTAAAATCAAAGTAGGCAA |
|  | 150112PeV-C8-F2 | ATGAGACAGTGATTTCTCCTATGCC |
|  | 150112PeV-C1-R1 | TGGTATTATAGCTGGGGCTCTTAAT |
|  | 150112PeV-C1-R2 | CCTGCTTTTACCAAGGTTTCTACTT |
|  | 150112PeV-C1-F1 | GTGTTGCTGAAACTGTCAGAGTTGT |
|  | 150112PeV-C1-F2 | AAAGAAGAAGAACTGGCAGAAGTCT |
|  | 150112PeV-C6-R1 | CAACCACATCCACAGCTGCTT |
|  | 150112PeV-C6-R2 | TAGTCTTCAACTTGTGAACCACCAA |
|  | 150112PeV-C6-F1 | CTGTGTAAATCTGACCCTGAAACAA |
|  | 150112PeV-C6-F2 | CAGCAAGCAATCCAATCAGAAC |
|  | 150112PeV-C9-R1 | TTGAAAAGGTCATCATGTAGGTCTG |
|  | 150112PeV-C9-R2 | ATGTAGGTCTGGGTGCTGTTCAT |
|  | 150112PeV-C9-F1 | CCAACAGTTAGAGCTCAGTGTAGCA |
|  | 150112PeV-C9-F2 | TAGCACATCTGATACACACGATTCC |
|  | 150112PeV-C13-R1 | TTTGTACCTTTCTAACCCTTCCAGT |
|  | 150112PeV-C13-R2 | GCTGTTTGTCAGTTTTTAAGTCCAA |
| RtAd-PV/SAX2015 | L2pestV16-F1 | TTCTTGGGAGAGAGCCCTATACA |
|  | L2pestV16-F2 | CCTATACATTGAGCTGGGTCCTAA |
|  | L2pestV8-R1 | CTCCACTAAAGAAAAGTCCGATCTC |
|  | L2pestV8-R2 | AAAAGTCCGATCTCTGCACTATAGG |
|  | L2pestV8-F1 | AGAACTCATTGAGATGATCACATGG |
|  | L2pestV8-F2 | ATACATGAACTGCTGACAATCTGGA |
|  | L2pestV13-R1 | GTATGCAGTTAAAAGCCATGCTG |
|  | L2pestV13-R2 | CACTACCATAACCAAGGGGAGATAC |
|  | L2pestV13-F1 | ACGGATACCAATATTGGTTTGACTT |
|  | L2pestV13-F2 | CCATTACATTGGGGGAGTAATAAAG |
|  | L2pestV7-R1 | CCTTTTGAGCCTGATATACTTTGTG |
|  | L2pestV7-R2 | TGCCTCAGGGTTGTACACTATACAC |
|  | L2pestV4-F1 | CTACAAAATACTCAAAGACCAGCCA |
|  | L2pestV4-F2 | ACCCTTCTACTTCTACGCAACTGAG |
|  | L2pestV12-R1 | TGCTGCTGTATGTAACAGTTCAAAG |
|  | L2pestV12-R2 | CAAAGATACAGGACATGATGGTTTC |
|  | L2pestV12-F1 | TGTAAAAAACTTCCTAGACCAGGCT |
|  | L2pestV12-F2 | ACAGATGAACTGTGTAAAGCAAACC |
|  | L2pestV10-R1 | TGCCACATTGTGCTCTTGTGA |
|  | L2pestV10-R2 | TGCATCAATGCGCCAATCT |
|  | 160126L2CV-F1 | GTGGGATGGATATAACGGTGAGA |
|  | 160126L2CV-F2 | GTGAGAACACCGTTATATTCGATGA |
|  | 160126L2CV-R1 | CCAACCTCTTTGCCTATAATAAGGT |
|  | 160126L2CV-R2 | AGTCTTCGCACGTATGCAAGAT |
| RtNe-PV/SC2014 | 1013L5PVC8-F1 | AAAGTTACACTGAGACTGCCCCA |
|  | 1013L5PVC8-F2 | ATGAAAGAGGGTTTAAGACAACACC |
|  | 1013L5PVC1-R1 | CTCTTCCTGATTAGTCCAGCAAAAC |
|  | 1013L5PVC1-R2 | TTACACCTTGCTTACAACCAGTTGT |
|  | 1013L5PVC1-F1 | TGCAGTAACATGCAGGTTTAACAG |
|  | 1013L5PVC1-F2 | GAAAACCAACATTGTGTTACAGGC |
|  | 1013L5PVC11-R1 | GGTCAGTATTTTGAAGGTCAAACCA |
|  | 1013L5PVC11-R2 | TCATATATTGCGCCCAGAATCTAG |
|  | 1013L5PVC11-F1 | TGATTCAATCATTGGACCGATG |
|  | 1013L5PVC11-F2 | CCTTGCGTGCCTATAAAAATCA |
|  | 1013L5PVC5-R1 | TCCCAGTGACGTGTTCAACACT |
|  | 1013L5PVC5-R2 | CTATATGCCCATCCAGTCTCAAAG |
|  | 1013L5PVC5-F1 | GGGCAGTAGTCCATTTAAAAAAGTG |
|  | 1013L5PVC5-F2 | GATTTCAAATGTGTCACAGTGGATG |
|  | 1013L5PVC2-R1 | GAAAGGCTTGTGTCTTTTTTCTAGG |
|  | 1013L5PVC2-R2 | TATCCTATGTATGTCATCAGGCCAC |
|  | 1013L5PVC2-F1 | TATCGGATGAGTTAACCCAACTCA |
|  | 1013L5PVC2-F2 | ACCCATCCCTTATATATTCCCAGA |
|  | 1013L5PVC6-R1 | CAGGTAAGCGATCAAAGGTTCA |
|  | 1013L5PVC6-R2 | AGGTTCAATCAAAGTGTGTAGGTCA |
|  | 1013L5PVC6-F1 | GGCTATGGCAAAAGGTAGAAGG |
|  | 1013L5PVC6-F2 | ATGTAGCAACTGTCTTGGTATCAGC |
|  | 1013L5PVC7-R1 | TGCTCTTGTGATGGGCAATTTA |
|  | 1013L5PVC7-R2 | TTGCATCAGAGCGCCTATCTT |
|  | 1013L5PVC7-F1 | ACAACTGAAAAACCTAGCATGCC |
|  | 1013L5PVC7-F2 | GGTACTGTTGAGAGGGGTTTCATAA |
|  | 1013L5PVC10-R1 | TTTGGGGTTTCCCTGCTTC |
|  | 1013L5PVC10-R2 | CTGCAAACCTTCTTGCCAGTT |
| RtNc-PV/SAX2015 | L4PESTV8-F1 | TGTGCCAAAGGTGGGTGAGT |
|  | L4PESTV8-F2 | TGTAACGACATTGACACATCTGGTA |
|  | L4PESTV10-R | TGTTATATCTGCACTGTATGGCCAC |
|  | L4PESTV10-F1 | CATCCACTGAACCAACTTCTAGTGT |
|  | L4PESTV10-F2 | ATTCCCACAAGTGTATACGTCAATG |
|  | L4PESTV6-R1 | TATGTGCATGTAGTTCTTGATGGTG |
|  | L4PESTV6-R2 | GCATGGGAGAGATTACAGTTTCATT |
|  | L4PESTV6-F1 | CGAAAAAGACAATTACTGGGGTC |
|  | L4PESTV6-F2 | CAATACATGATCAAGGATGGTTACC |
|  | L4PESTV1-R1 | TCTACTACCTTTGGAATTATGGCTG |
|  | L4PESTV1-R2 | CCTGCTCTCACCAAAGTTTCTACTT |
|  | L4PESTV1-F1 | AGACAGTTAGAGTGGTTGCAATGAC |
|  | L4PESTV1-F2 | GACATAAAGGAAGAAGAATTGGCTG |
|  | L4PESTV7-R1 | GTCACCGTAACCATTTGTGTAGATG |
|  | L4PESTV7-R2 | GCAGATATTAACACAGTTGCCACAT |
|  | L4PESTV7-F1 | ACAATGCTTGAACCTTTGTTGAG |
|  | L4PESTV7-F2 | TTGTTGAGTTATCTGCCATACGC |
|  | L4PESTV2-R | GGTATTGCAGTCTCATAGTATTGCG |
